# BARD1: A Friend or Foe in Pancreatic Ductal Adenocarcinoma?

**DOI:** 10.3390/ijms26189041

**Published:** 2025-09-17

**Authors:** Lily Zekavat, Aditi Jain

**Affiliations:** 1 Sidney Kimmel Medical College, Thomas Jefferson University, Philadelphia, PA 19107, USA; 2 Jefferson Pancreas, Biliary and Related Cancer Center, Department of Surgery, Thomas Jefferson University, Philadelphia, PA 19107, USA

**Keywords:** BARD1, BRCA1-BARD1, PDAC, double-strand break repair, review

## Abstract

Pancreatic ductal adenocarcinoma (PDAC) is an aggressive solid malignancy with poor overall prognosis and limited response to standard treatments. Growing interest in the modulation of DNA repair mechanisms, including the homologous recombination (HR) repair pathway, has opened new avenues for therapeutic development. BARD1 (BRCA1-Associated RING Domain 1) plays a complex role in tumor biology, functioning either as a tumor suppressor or as an oncogenic driver, depending on isoform expression, cellular context, and regulatory environment. In this review, we examine the dual roles of BARD1, focusing on its regulation and paradoxical activities in PDAC. We summarize evidence that BARD1 and BARD1 isoforms differentially affect DNA repair, apoptosis, and drug resistance and evaluate the therapeutic potential of targeting BARD1 and other DNA damage response (DDR) proteins. We also review ongoing clinical trials and investigational agents designed to exploit DDR vulnerabilities in PDAC. Together, these insights highlight BARD1’s context-dependent roles in PDAC and support continued efforts to profile BARD1 isoforms, clarify their functions, and leverage DDR pathways through precision oncology approaches.

## 1. Introduction

Pancreatic ductal adenocarcinoma (PDAC) is the most common malignant tumor of the pancreas, accounting for over 90% of pancreatic cancer cases. PDAC is predominantly driven by inactivating mutations in genes that have historically posed significant challenges as drug targets, including *KRAS*, *CDKN2A*, *TP53*, and *SMAD4* [1,2,3,4]. Disease prognosis remains poor, largely due to typically late-stage diagnosis and resistance to conventional therapies [5]. While several clinical trials have been conducted over the past decade, most have failed to yield significant advances in treatment outcomes [6]. The current 5-year overall survival rate stands at just 13%, highlighting the urgent need for more effective and targeted therapeutic strategies [7].

PDAC is characterized by extensive genomic instability, frequently driven by defects in key DNA damage response (DDR) pathways critical for preserving genomic integrity [3,8,9,10,11]. Among the key regulators of DDR is BRCA1-Associated RING Domain 1 (BARD1), which, both independently and in complex with its obligate binding partner, BRCA1, recruits and stabilizes DNA repair machinery at damage sites and coordinates key steps of the homologous recombination (HR) repair pathway [12,13]. Mechanistically, BARD1 and BRCA1 interact through their respective N-terminal RING-binding domains, forming a heterodimer that is recruited to sites of DNA double-stranded breaks. This complex promotes end resection of double-stranded breaks, generating 3′-single-stranded DNA overhangs required for strand invasion, and facilitates the recruitment of downstream HR factors, including PALB2, BRCA2, and RAD51 [14,15,16,17,18,19]. The ability of BARD1 to engage RAD51 is particularly important for initiating strand invasion, and disruption of this interaction impairs HR efficiency [17]. Loss of BARD1 function, whether through gene silencing or mutations in domains critical for BRCA1 or RAD51 binding, undermines HR activity and promotes genomic instability. Such impairment may give rise to a “BRCAness” phenotype, in which cells become reliant on alternative, error-prone repair pathways and accumulate additional DNA damage [20].

Therapeutically, BARD1 deficiencies may represent an exploitable vulnerability. Tumors with HR repair defects are hypersensitive to agents that induce DNA damage, such as platinum-based chemotherapies and poly (ADP-ribose) polymerase inhibitors (PARPis) through synthetic lethal interactions. PARP inhibition impairs the repair of single-stranded breaks; when unresolved, these lesions cause replication fork collapse, generating double-stranded breaks that are lethal in cells lacking functional HR repair [21,22]. The PARPi Olaparib is the first and only member of this therapeutic class to receive approval for the treatment of *BRCA1/2*-mutant PDAC based on Phase III clinical trial data demonstrating improved progression-free survival compared to placebo (7.4 vs. 3.8 months; hazard ratio 0.53, *p* = 0.004) in the maintenance setting [23]. However, broader clinical use of Olaparib is hampered by both inherent and acquired resistance mechanisms, including restoration of HR proficiency [24]. Nevertheless, extending these insights to the significant proportion of PDACs lacking *BRCA1/2* could be valuable, and personalized therapeutic approaches targeting DDR pathways hold considerable promise.

In this review, we examine the multifaceted role of the DDR protein BARD1 in PDAC, with a focus on its complex regulatory mechanisms and the dual tumor-suppressive and oncogenic functions. We further discuss the potential of BARD1 as a promising therapeutic target in DNA repair-based strategies for the treatment of PDAC and highlight ongoing clinical efforts targeting DDR mechanisms.

## 2. Overview of BARD1

**Figure 1:**
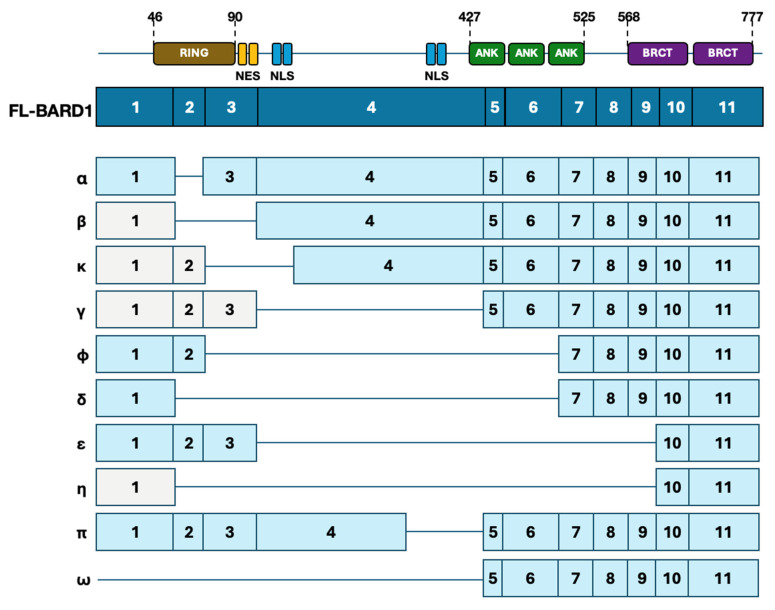
Diagram showing the structure of full-length BARD1 (FL-BARD1) and select isoforms. Domain boundaries are annotated with their approximate amino acid positions. Key functional motifs and corresponding exons are highlighted. FL-BARD1 is depicted in dark blue. Isoforms of BARD1 are depicted in light blue. Non-coding exons of isoforms are depicted in light gray. NES: nuclear export signal; NLS: nuclear localization signal; ANK: ankyrin repeat; BRCT: BRCA1 C-terminal.

The human *BARD1* gene encodes a 777-amino acid protein located on chromosome 2q34-35 and was originally identified as a binding partner of BRCA1 [25]. Structurally, the full-length (FL) BARD1 protein consists of 11 exons and contains a RING (Really Interesting New Gene) finger domain at the N-terminus, three ankyrin repeat (ANK) domains, and two BRCT (BRCA1 C-terminus) domains, along with two nuclear localization signals (NLSs) and one nuclear export signal (NES) (Figure 1) [26,27,28,29,30]. Heterodimerization with BRCA1 is critical for the complex’s E3 ubiquitin ligase activity [31]; while the BRCA1 RING domain can bind E2~Ub conjugates independently, BARD1 enhances ligase function and stabilizes the complex [32]. Additionally, BARD1 binding masks NES motifs present in the BRCA1 protein, promoting nuclear retention of the complex necessary for HR proficiency [33]. Despite the well-characterized roles of BARD1 in genome maintenance, BARD1 isoforms that lack the BRCA1-interacting domain or other critical regions may lose tumor-suppressive capabilities and instead contribute to tumorigenesis. The following sections will explore the contrasting roles of BARD1 and BARD1 isoforms in greater detail.

## 3. Tumor Suppressor Effects of BARD1

The E3 ubiquitin ligase activity of the BRCA1-BARD1 heterodimer is essential to its tumor suppressor function, enabling the complex to polyubiquitinate various substrates and regulate diverse cellular processes. Mechanistically, BRCA1-BARD1 interacts with UbcH5 E2 ubiquitin-conjugating enzymes and catalyzes the formation of K6-, K48-, or K63-linked ubiquitin chains of the target protein [12]. Among the best characterized substrates of BRCA1-BARD1 is nucleosomal histone H2A, reflecting the role of the complex in facilitating DNA double-stranded break repair [34]. Additional substrates include estrogen receptor α (ERα), proliferating cell nuclear antigen, cyclin B1, and CtBP-interacting protein, indicating broader roles in transcriptional regulation and cell cycle control [34,35,36]. Loss of E3 ligase activity leads to HR deficiency (HRD), a phenotype similar to that caused by the cancer-associated BRCA1 C61G mutation, which weakens the BRCA1-BARD1 interaction and disrupts BRCA1’s binding to E2 enzymes, thereby abolishing the ligase function [37,38,39].

Independent of BRCA1 complex formation, BARD1 alone is essential for maintaining genomic stability. Complete loss of BARD1 leads to chromosomal abnormalities and embryonic lethality between embryonic days E7.5 and E8.5 in mouse models [40]. In BRCA1-deficient contexts, BARD1 can suppress tumorigenesis by promoting p53-dependent apoptosis. BARD1 binds and stabilizes both unphosphorylated and Ser15-phosphorylated forms of p53, competing with p53’s negative regulator, MDM2, in the nucleus; accordingly, exogenous or genotoxic stress-induced overexpression of BARD1 has been shown to stabilize the p53 protein and trigger apoptosis with classical features of programmed cell death [41,42]. Additionally, BARD1 can translocate to the mitochondria during apoptosis, where BARD1 enhances p53 phosphorylation by DNA-dependent protein kinase [43]. BRCA1 binding interferes with the nuclear export of BARD1, thereby limiting the pro-apoptotic function of BARD1 and regulating the subcellular localization of the BRCA1-BARD1 complex [44].

In addition to established roles in HR and apoptosis, BARD1 also contributes to genomic stability by regulating mitotic spindle assembly. BARD1 has been identified as a spindle assembly factor activated by RanGTP, a small GTPase critical for proper mitotic spindle formation. Studies in Xenopus egg extracts and mammalian cells demonstrate that BARD1 localizes to spindle poles in a manner dependent on RanGTP gradients and regulated by importin β. This interaction reflects a Ran-dependent pathway in which the release of BARD1 from importin complexes enables participation in microtubule nucleation and stabilization during mitosis [45].

Both germline and somatic BARD1 mutations have been described in pancreatic cancer, supporting BARD1’s role in tumor suppression; as such, BARD1 is currently included in germline mutation testing panels, along with other DNA repair genes [9,46,47]. However, the direct impact of these mutations on BARD1’s tumor suppressor function and their potential prognostic value requires further clarification. In the following section, we will review mechanisms that influence the tumor suppressor functions of BARD1, including select mutational events that affect isoform expression, protein stability, and DNA repair activity.

## 4. Mechanisms Affecting Tumor-Suppressive Activity of BARD1

### 4.1. Genomic Alterations

Several germline variants in BARD1, associated with elevated risk for breast, ovarian, pancreatic, and other cancers, are mappable to highly conserved domains [39,48,49,50]. Mutations within the ANK domains, which mediate protein–protein interactions, may impair DNA repair, pre-mRNA splicing, or interaction with BRCA1 [42,43,51]. Deletions involving exons 2–6, which encode most of the RING and ANK domains, abolish BRCA1 binding entirely and compromise complex formation [52]. Similarly, pathogenic variants in the highly conserved BRCT domains, which typically facilitate recruitment of the BRCA1-BARD1 complex to sites of DNA damage, impair DNA repair activity and disrupt tumor-suppressive function [53,54]. Several frameshift and missense mutations in or near the BRCT domains—such as Cys645Arg, Val695Leu, and Ser761Asn—have been identified as cancer-predisposing alterations [53]. However, the role of isolated RING domain mutations in BARD1 remains more controversial. For example, the RING domain mutation BARD1^R99E^ was initially reported to impair HR-driven DNA double-stranded break repair and confer sensitivity to genotoxic agents, including topoisomerase inhibitors, ionizing radiation, and Olaparib [55]; however, a separate study later found that re-expression of this mutant allele in BARD1-depleted cells restored resistance to PARPis, implying that the RING domain may be dispensable for HR proficiency or tumor suppression [56].

Mutations outside BARD1’s canonical functional domains may also impact tumor suppressor activity. For example, the Gln564His variant, located between the ANK and BRCT domains, reduces BARD1’s interaction with Cleavage Stimulating Factor 50, a protein involved in RNA processing and p53-mediated apoptosis [48,57]. BARD1 interacts with p53 through both the ANK-BRCT linker region and the BRCT domain, stabilizing p53 and promoting apoptotic signaling. Disruption of this interaction, including through mutations in the linker region, has been associated with defective apoptosis and contributes to the pathogenesis of breast, ovarian, and uterine cancers [43,51].

Mechanistically, many pathogenic BARD1 variants impair HR, underscoring a critical role in the impairment of BARD1’s tumor suppressor functions. In a pan-cancer analysis of over 10,000 tumors, Adamovich et al. identified 76 potentially pathogenic BARD1 missense variants, of which 16 were functionally deficient in HR. These HR-deficient variants sensitized cells to cisplatin and ionizing radiation, consistent with compromised DNA repair. Notably, several variants within the ANK and BRCT domains disrupted HR activity beyond their known roles in protein interaction, suggesting additional, unrecognized functions in DNA repair. Conversely, other variants associated with high loss of heterozygosity, such as Ser339Thr, Thr343Ile, and Val523Ala, retained HR function, suggesting that loss of heterozygosity is not a reliable proxy for predicting functional inactivation of BARD1 [54].

Beyond rare pathogenic mutations, single-nucleotide polymorphisms (SNPs) in *BARD1* have also emerged as important regulators of cancer susceptibility, particularly highlighted in neuroblastoma, nephroblastoma, and gynecologic malignancies [58,59]. Briefly, SNPs in *BARD1* are among the most consistently identified neuroblastoma-susceptibility genes across multiple ethnic groups [60], although cancer susceptibility varies depending on the genomic context and the functional consequences of these variants on *BARD1* mRNA expression. For example, genome-wide association studies in neuroblastoma suggest that SNPs linked to elevated expression of FL-BARD1 correlate with reduced susceptibility to malignancy. Similarly, in breast cancer, some studies have found that BARD1 polymorphisms were associated with a significantly decreased breast cancer risk; however, more research is needed to understand how SNP-altered gene expression patterns affect tumor behavior [61]. We will explore the common variants of BARD1 in greater detail later in the review.

In pancreatic cancer specifically, several rare *BARD1* variants have been identified in both familial and sporadic cases. These include a truncating mutation (c.632T > A; p.Leu211 *) in a patient with a prior family history of malignancy and a nonsense variant (c.1921C > T; p.Arg641 *; rs587781948) also associated with neuroblastoma risk [46,47]. Both variants introduce premature stop codons and are predicted to impair FL-BARD1 function, though their effects on oncogenic isoform expression remain unclear. While germline loss-of-function variants have been reported across several tumor types, with over 4000 identified in the National Center for Biotechnology Information ClinVar database [62,63,64], most *BARD1* variants are classified as either “benign” or of “uncertain significance” due to limited clinical and functional data [46,47,65,66,67,68,69,70,71,72,73]. Additional research is needed to define their biological and clinical relevance. A list of select known *BARD1* mutations in pancreatic cancer is shown in Table 1.

### 4.2. Post-Translational Modulation of BARD1

The cellular levels of BARD1 and its interaction with BRCA1 are critical for the heterodimeric function of the BRCA1-BARD1 complex in HR repair and tumor suppression. BARD1 protein levels are tightly regulated through multiple mechanisms and signaling pathways and are maintained at a steady state throughout the cell cycle, primarily through association with BRCA1 [74]. Post-translational modifications influence BARD1’s stability, localization, and functional interactions with other proteins. The key post-translational modifications of BARD1 include ubiquitination, phosphorylation, and SUMOylation events. BARD1 protein stability is enhanced by phosphorylation, with hyperphosphorylated forms of BARD1 observed in mitotic cells [75]. In addition to phosphorylation, SUMOylation has emerged as a key post-translational modification regulating BARD1 function. Proteomic analysis by Hendriks et al. revealed that replication stress induced by hydroxyurea led to significant increases in SUMOylation of both BRCA1 and BARD1, implicating SUMOylation as a mediator of DDR activation in response to sustained replication stress. These findings are consistent with prior mechanistic studies indicating that SUMOylation enhances BRCA1–BARD1 E3 ligase activity and promotes recruitment to DNA double-stranded breaks through SUMO-targeted ubiquitin ligases [76,77,78]. Additional regulators, including deubiquitinating enzymes and E3 ubiquitin ligases, may also influence BARD1 stability; however, regulatory mechanisms remain incompletely understood. Below, we summarize the known protein degradation pathways involved in the regulation and stabilization of BARD1.

Several E3 ligases have been identified as regulators of BARD1 and may play complementary roles in BRCA1-BARD1 complex modulation. Wu et al. demonstrated that HERC2, an E3 ligase implicated in DNA damage repair, ubiquitinates BRCA1 in a BARD1-independent manner. In this model, unbound BRCA1 is degraded during the S phase via the HECT domain of HERC2. Knockdown of HERC2 stabilizes BRCA1 levels and indirectly increases BARD1 levels, likely due to reciprocal stabilization of the complex. Therefore, targeted inhibition of HERC2 may enhance BARD1 stability and thereby promote tumor suppression [79]. Similarly, HUWE1, another E3 ligase, decreases BRCA1 stability via ubiquitination. Although HUWE1 does not directly affect BARD1 ubiquitination, BRCA1 stabilization following HUWE1 knockdown indirectly increases BARD1 levels [80]. The F-box protein FBXO44 also ubiquitinates BRCA1 and co-immunoprecipitates with both BRCA1 and BARD1, suggesting potential interaction with BARD1 independent of BRCA1 and a possible role in regulating BARD1 degradation [81].

Other E3 ligases, termed Cullin–RING ligases (CRLs), require DDB1- and CUL4-associated factors (DCAFs) for substrate recognition; several DCAFs have been implicated in the regulation of BARD1 stability. DCAF8L2, as described by Deng et al., acts as a negative regulator of BARD1 stability through direct binding of the BARD1 RING domain, thereby disrupting BRCA1-BARD1 complex stability [82]. A related X-linked DCAF, DCAF8L1, plays a similar role, complexing with CRL4 and directing CRL4 activity toward ubiquitination and proteasomal degradation of both BARD1 and BRCA1 [83]. As enrichment of both DCAF8L1 and DCAF8L2 is observed in certain human breast cancer cells, these factors may promote carcinogenesis via negative regulation of BRCA1 and BARD1. The role of DCAFs in mediating BARD1 degradation in pancreatic cancer remains to be explored.

The anaphase-promoting complex/cyclosome, an evolutionarily conserved E3 ligase regulating cell cycle progression, also promotes ubiquitin-mediated degradation of BARD1. Given the previously identified role of BARD1 in regulating Ran-dependent mitotic spindle assembly, anaphase-promoting complex/cyclosome-mediated degradation of BARD1 may represent a mechanism by which the cell ensures genomic integrity and faithful chromosome segregation [84].

Apart from E3 ligases, other proteins, including Sirtuins, have also been implicated in BARD1 regulation. SIRT2, in particular, has been shown to deacetylase BARD1 at conserved lysine residues within the RING domain, promoting stability of the BRCA1-BARD1 complex and enabling tumor-suppressive function [85]. The role of Sirtuins in cancer pathogenesis, however, is complex and context-dependent, with additional reported roles in apoptotic inhibition, proliferation, and acquisition of genetic mutations [86,87,88]. As such, depending on cellular context, Sirtuins may support HR indirectly via stabilization of BARD1, or conversely, contribute to tumor progression and therapy resistance.

### 4.3. Promoter Methylation

While promoter methylation of BRCA1 is frequently observed across cancers, BARD1 promoter methylation is comparatively rare, suggesting that preservation of BARD1 expression may contribute to its tumor suppressor function [89,90,91,92]. A recent study investigating adaptive resistance to anti-VEGF antibody therapy in ovarian cancer found that, in anti-VEGF antibody-resistant tumors, BARD1 expression was downregulated and associated with increased promoter methylation. Treatment with the DNA methyltransferase inhibitor 5′-azacytidine in combination with a murine anti-VEGF antibody significantly restored BARD1 expression and reduced tumor burden in multiple in vivo models. Notably, BARD1 expression inversely correlated with tumor weight, and BARD1 knockdown promoted angiogenic signaling pathways and therapeutic resistance [93]. Together, these findings suggest that the absence of BARD1 promoter methylation, and the consequent maintenance of BARD1 expression, may play a tumor-suppressive role in certain cancer contexts.

In a separate study by Lubecka et al., BARD1 expression was found to be upregulated in hepatitis B virus-negative patients at risk for developing hepatocellular carcinoma. This increase was associated with significant hypomethylation of the *BARD1* gene, with a 13.3% reduction in DNA methylation compared to healthy controls. These findings suggest that hypomethylation of the *BARD1* gene may serve as a predisposing factor in the development of hepatocellular carcinoma in hepatitis B-negative patients [94].

Beyond DNA methylation, other modifications could also influence BARD1’s tumor-suppressive role. Studies in pluripotent stem cells have identified H3K36me3, an activating histone mark, as a determinant of BARD1 alternative splicing, suggesting that chromatin state may govern isoform-specific transcription of the *BARD1* gene [95]. While this mechanism remains to be validated in cancer, this finding raises the possibility that histone marks not only affect BARD1 expression levels but may also drive isoform heterogeneity.

## 5. Oncogenic Effects of BARD1

The oncogenic potential of BARD1 is largely attributed to the truncated or alternatively spliced transcript variants, which may act in a dominant-negative manner or acquire novel functions. To date, at least 19 isoforms of the BARD1 protein have been identified, including BARD1α, BARD1β, BARD1γ, BARD1δ, BARD1ε, BARD1η, BARD1κ, BARD1π, BARD1ϕ, and BARD1ω [96]. Some of these are annotated in the Ensembl genome database, while others are listed in the NCBI Reference Sequence (RefSeq) database. The expression of certain BARD1 isoforms is upregulated in a wide range of malignancies, including breast, ovarian, uterine, lung, neuroblastoma, melanoma, leukemia, and colon cancers [97,98,99,100,101,102,103,104,105]. However, their precise cellular functions appear to be both isoform- and tissue-specific. Select isoforms and their structural features are presented in Figure 1.

### 5.1. Isoform-Specific Oncogenic Effects

#### 5.1.1. BARD1β and BARD1δ Isoforms

Several groups have investigated the BARD1β isoform, a 680-amino acid (75 kDa) protein, which is overexpressed in colorectal cancer, lung cancer, and high-risk neuroblastoma [98,100,103]. In neuroblastoma cells, Bosse et al. demonstrated that BARD1β knockdown significantly increased caspase-3 and -7 activity, indicating enhanced apoptotic signaling; notably, however, BARD1β silencing did not change cellular levels of Ser15-phosphorylated p53 nor sensitize cells to PARPis, suggesting that the effects of BARD1β on apoptosis were independent of FL-BARD1/p53 phosphorylation and HR deficiency [106]. Parallel studies demonstrated that BARD1β knockdown also led to reduced levels of both Aurora kinase A and B, mitotic kinases whose expression promotes chromosomal segregation errors in cancer, and that parallel knockdown of either kinase or BARD1β showed comparable growth inhibition. Furthermore, in non-transformed NIH-3T3 murine fibroblasts, overexpression of BARD1β, but not FL-BARD1, conferred resistance to apoptosis and enabled proliferation under low-serum conditions, a phenotype fully reversed by RNAi targeting of BARD1β and partially reversed by targeting Aurora A or B kinase [106]. Taken together, these data implicate an oncogenic function of BARD1β through both promotion of chromosomal segregation errors and FL-BARD1-independent evasion of apoptosis.

BARD1δ, a structurally distinct, 307-amino acid (35 kDa) isoform lacking exons 2–6, is likewise overexpressed across various tumor types, including breast and ovarian cancer, particularly in aggressive subtypes, such as clear cell carcinoma [89,102,104,106,107]. Unlike BARD1β, which promotes mitotic dysregulation and apoptosis evasion, BARD1δ directly disrupts telomere stability through aberrant, high-affinity binding to telomere-associated proteins, leading to their sequestration and subsequent chromosomal instability. While this telomeric dysfunction induces mitotic arrest in non-transformed cells, cancer cells often bypass checkpoint arrest, allowing continued proliferation [108].

**Figure 2:**
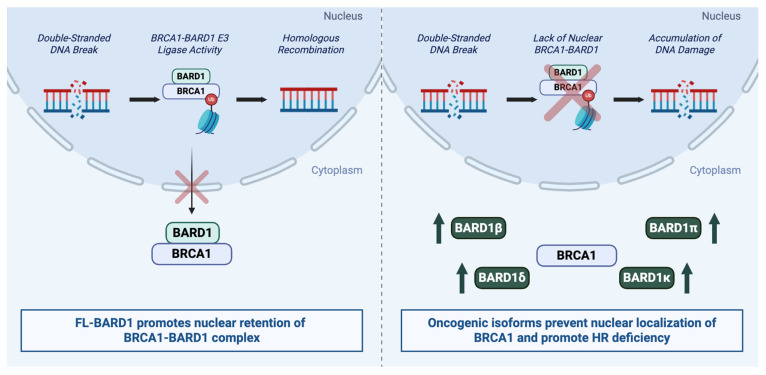
Comparison of FL-BARD1 and BARD1 isoforms in DNA damage response pathways. FL-BARD1 binds to BRCA1 via the RING domain and inhibits BRCA1 nuclear export. The BRCA1-BARD1 complex, localized to the nucleus, ubiquitinates histones at sites of DNA damage, a step crucial for DNA damage response (**left image**). BARD1 isoforms lacking the RING domain do not bind BRCA1 and could prevent the nuclear retention of BRCA1 in tumor cells (**right image**). HR: homologous recombination.

Additionally, BARD1β and BARD1δ may promote oncogenesis by acting as dominant-negative disruptors of both BRCA1-BARD1 E3 ligase activity and nuclear localization of BRCA1, as both isoforms lack the N-terminal RING domain required for interaction with BRCA1. In ER^+^ breast cancer cells, both BARD1β and BARD1δ interfere with FL-BARD1-mediated degradation of ERα, a known target of BRCA1-BARD1 ubiquitin ligase activity. While FL-BARD1 facilitates ERα turnover, overexpression of BARD1β or BARD1δ impairs this process, resulting in sustained ERα stability and signaling. Co-immunoprecipitation assays demonstrate that although BARD1β and BARD1δ retain the ability to bind ERα, they fail to associate with BRCA1, further supporting their dominant-negative effect. This disruption of hormone receptor regulation may contribute to oncogenic signaling in hormone-responsive cancers [99]. In support of this dominant-negative mechanism, additional data in colorectal cancer cells show that exogenous overexpression of BARD1β increases cytoplasmic retention of BRCA1, impairs HR, and sensitizes cells to PARPis by disrupting nuclear BRCA1 function [98]. Illustrative comparison of FL-BARD1 and certain BARD1 isoforms is depicted in Figure 2.

#### 5.1.2. Additional Isoforms and Paradoxical Roles

Beyond BARD1β and BARD1δ, other isoforms have also been implicated in cancer. The φ isoform has been associated with poor prognosis in ovarian and breast cancer patients [104,109], while the ω isoform is upregulated in acute myeloid leukemia, where BARD1ω induces mitotic errors and nuclear enlargement, consistent with heightened genomic instability [105]. In non-small cell lung cancer tissue samples, Zhang et al. found that overexpression of BARD1π and BARD1κ isoforms, lacking the nuclear localization signal and BRCA1 interaction domain, respectively, induced BRCA1 mislocalization to the cytoplasm, implying deficiency in HR and suggesting that additional isoforms beyond BARD1β and BARD1δ may exert a dominant-negative effect on nuclear localization [103].

Paradoxically, some findings challenge the consistently oncogenic role of BARD1 isoforms. In colon cancer, BARD1δ and ϕ were reported to exert tumor-suppressive functions in at least one context [107]. With respect to nuclear localization, in triple-negative breast cancer cell lines, one study observed high BARD1β mRNA levels alongside persistent nuclear BRCA1 localization [110]. Furthermore, a separate study of primary breast cancer tissues found that while BRCA1 and BARD1 predominantly colocalize in the cytoplasm, the α and β isoforms are not overexpressed in most samples, suggesting that isoform expression may not be the primary driver of BRCA1-BARD1 mislocalization in certain cancers [101]. These discordant roles emphasize that BARD1 isoform function is highly context-dependent and shaped by isoform-specific structure and tumor type, and, therefore, requires careful evaluation of its cell-specific activities.

## 6. Mechanisms Affecting Oncogenic Activity of BARD1

### 6.1. MicroRNA-Mediated Post-Transcriptional Upregulation of BARD1 Isoforms

Emerging evidence highlights the role of post-transcriptional regulation in driving oncogenic BARD1 isoform expression across cancer types. Pilyugin and Irminger-Finger et al. identified long non-coding RNAs and microRNAs as key regulators of BARD1 isoform expression, proposing a model of post-transcriptional control that contributes to isoform dysregulation in cancer [111]. In particular, the authors characterized a non-coding transcript termed BARD1 9’L, originating near an estrogen response element within intron 9 of the BARD1 gene. Functionally, BARD1 9’L may act as a competing endogenous RNA, sharing microRNA-binding elements with protein-coding BARD1 transcripts and thereby sequestering microRNAs that would otherwise target oncogenic BARD1 isoforms for degradation. Notably, FL-BARD1 possesses a longer 3’ untranslated region than many isoforms, making the full-length form more vulnerable to microRNA-mediated downregulation [111]. This structural difference between FL- and BARD1 isoforms may shift expression balance in favor of shorter, oncogenic isoforms, particularly in promoting malignancy. Furthermore, single-nucleotide polymorphisms associated with neuroblastoma risk, such as rs7585356, were shown to alter microRNA binding within the BARD1 3’ untranslated region (3’UTR), further linking genetic variation to isoform-specific expression control [111]. Supporting this model, a separate study by Lepore et al. found that the histone deacetylase inhibitor vorinostat reduced *BARD1* mRNA levels via upregulation of miRNAs miR-19a and miR-19b, which bind directly to the BARD1 3’ untranslated region and cause degradation of the *BARD1* mRNA [105]. Reduced expression of such miRNAs, coupled with overexpression of BARD1 isoforms, may contribute to tumor progression, an area that remains largely unexplored in pancreatic cancer.

### 6.2. RNA-Binding Protein (RBP) Mediated Stabilization of BARD1 Isoforms

A recently described mechanism of BARD1 regulation is via Human Antigen R (HuR/ELAVL-1), an RNA-binding protein that modulates the stability of mRNAs encoding pro-survival and pro-resistance genes. Our group previously published that HuR regulates BARD1 expression in pancreatic cancer cells through a non-canonical mechanism involving both pre-mRNA and mature mRNA transcript levels [112,113,114,115]. This regulation appears to be isoform-specific, as HuR inhibition results in consistent downregulation of BARD1 α, δ, and φ isoforms. Notably, HuR shows increased binding affinity to BARD1δ and φ transcripts following treatment with Olaparib, suggesting that HuR may promote upregulation of oncogenic BARD1 isoforms in response to DNA-damaging agents [113].

### 6.3. BARD1-Mediated Oncogenic Transcriptional Networks

Although BARD1 isoforms have been implicated in oncogenesis, often without mechanistic insight, our recent findings reveal that BARD1 supports malignant transcriptional programs in PDAC by sustaining MYC-mediated transcriptional activity. This, in turn, promotes the expression of genes critical for tumor cell growth and survival [116,117]. We observed BARD1 overexpression at both the mRNA and protein levels across multiple PDAC cell lines in both cell culture and tissue samples, independent of KRAS mutation subtype. Further, BARD1 silencing was found to significantly reduce colony formation, cell proliferation, and invasion in vitro and slow tumor growth in vivo in mouse xenografts [117]. Mechanistically, BARD1 appears to regulate c-MYC at the protein level, as BARD1 knockdown reduced c-MYC protein expression without affecting mRNA levels. Our finding that BARD1 promotes c-MYC protein stability was further supported by the strong correlation observed between BARD1 and c-MYC protein levels. Notably, treatment with a proteasome inhibitor rescued c-MYC protein levels in BARD1-silenced cells, indicating that BARD1 may stabilize c-MYC by inhibiting c-MYC degradation through the ubiquitin–proteasome pathway. Together, these findings identify BARD1 as a key regulator of c-MYC stability, suggestive of a previously unrecognized oncogenic role for BARD1 in PDAC [117].

## 7. Role of BARD1 in Therapeutic Resistance

Chemotherapy resistance is a major driver of disease relapse and poor clinical outcomes across malignancies, including PDAC. One well-characterized mechanism of resistance involves the upregulation of DNA repair genes, which enables tumor cells to evade the cytotoxic effects of chemotherapeutic agents. This phenomenon has been widely documented in cisplatin-resistant tumors, in which elevated expression of multiple DNA repair genes correlates with poor therapeutic response and reduced patient survival [118,119]. DNA repair pathways also underlie resistance to radiation, underscoring their relevance as therapeutic targets [120,121].

In this context, dysregulation of BARD1 may contribute to chemoresistance across malignancies. In PDAC, analysis of patient-derived, Gemcitabine-treated organoids revealed that elevated BARD1 expression was significantly associated with progressive disease [122]. Likewise, pancreatic cancer stem cells isolated from human tumors also display increased expression of the binding partner of BARD1, BRCA1, enhancing their capacity to survive Gemcitabine treatment [123]. In breast cancer, tamoxifen-resistant cell lines express significantly higher levels of both BRCA1 and BARD1, diminishing the cytotoxic impact of chemotherapy [124].

Isoform-specific regulation of BARD1 also appears to influence cellular responses to chemotherapy. In MCF-7 breast cancer cells, which retain functional BRCA1, Marzec et al. demonstrated that overexpression of BARD1ω significantly reduced apoptosis following treatment with cisplatin when compared to cells expressing wild-type FL-BARD1. While FL-BARD1 and most variants enhanced cisplatin-induced apoptosis, BARD1ω consistently conferred resistance, suggesting an anti-apoptotic function [109]. BARD1β showed a trend toward reduced apoptosis at baseline and low-dose treatment, though this was not significant at higher concentrations. Conversely, the BARD1φ isoform was more responsive to cisplatin than FL-BARD1, indicating isoform-specific modulation of chemosensitivity. In HCC1937 cells, which are BRCA1-deficient, BARD1ε conferred resistance to cisplatin compared to FL-BARD1 [109]. Given that BRCA1 status influences the composition and function of BARD1-dependent intracellular apoptotic machinery, the elevation of specific BARD1 isoforms in response to chemotherapy may reflect differences in the mechanisms of apoptotic induction.

Our research has previously found that treatment of PDAC cells with either Olaparib or a platinum-based chemotherapy (oxaliplatin) leads to increased BARD1 expression, while transient silencing of BARD1 increases cellular sensitivity to these therapies. Further analysis revealed that this upregulation occurs via a post-transcriptional mechanism mediated by the pro-survival RNA-binding protein HuR [113]. Whether FL-BARD1 or BARD1 isoforms contribute to acquired resistance following chronic treatment with chemotherapy or radiotherapy remains to be determined.

In addition to a role in chemotherapy resistance, overexpression of DNA repair proteins has also been associated with immune checkpoint inhibitor resistance. Inhibiting DNA repair proteins, such as RAD51, PARP1, and mismatch repair components, has been shown to enhance anti-tumor immunity and improve response to immune checkpoint blockade [125,126,127]. Targeting DNA damage response proteins, such as BARD1, may thus represent a promising strategy to sensitize immune “cold” tumors, like PDAC, to immunotherapies.

## 8. Clinical Trials with DNA Damage Response (DDR) Agents

Several experimental and approved agents targeting DNA repair pathways are currently under investigation for the treatment of PDAC (Table 2). Among these, the PARPi Olaparib, approved in 2014 for germline *BRCA1/2*-mutated ovarian cancer and in 2019 for *BRCA1/2*-mutated PDAC, was the first agent to directly exploit DNA repair deficiencies in cancers [128]. By inhibiting single-stranded DNA break repair, Olaparib promotes the accumulation of DNA damage in repair-deficient tumor cells, inducing synthetic lethality, while sparing cells with intact repair mechanisms [21,22]. Several clinical trials are currently underway evaluating the efficacy of Olaparib and other PARPis in PDAC, either as monotherapies or in combination with other chemotherapeutic and immune-modulatory agents, as listed in Table 2 [129,130,131,132,133]. Efficacy signals for various DDR agents, including PARPis, are primarily seen in DNA repair-deficient cancers, and deficiencies in these pathways should be explored as targets for DDR agents. Despite being effective in different settings of DNA repair-deficient backgrounds, DDR agents display several on- and off-target toxicities. The most common toxicities are hematological and gastrointestinal toxicities seen in the majority of patients, while less frequent but potentially life-threatening (e.g., myelodysplastic syndromes) toxicities are also seen. Caution is warranted in the use of combinatorial therapeutic approaches, where the addition of several DDR-targeted agents should be properly managed to minimize adverse events of these drugs.

Beyond Olaparib, three additional PARPis—Talazoparib, Niraparib, and Rucaparib—have received approval in a range of cancer types and are currently being evaluated for the treatment of PDAC. Talazoparib, initially approved as a monotherapy in *BRCA1/2*-mutated breast cancer and in combination with enzalutamide for HR-deficient prostate cancer, is currently under Phase II investigation for patients with solid tumors harboring DDR gene mutations, including BARD1 [134]. Previous efforts to evaluate Talazoparib use in PDAC included the JAVELIN-PARP-MEKi study, in which enrolled patients received treatment of either Talazoparib plus binimetinib, a MEK inhibitor, or binimetinib plus avelumab, a PD-L1 inhibitor. Plans to evaluate a triplet combination consisting of all three drugs were abandoned, however, as dose-limiting toxicities associated with binimetinib resulted in the trial’s early termination. Among the thirteen PDAC patients treated with Talazoparib and binimetinib, no objective responses were observed, although three patients showed stable disease [135].

Niraparib, approved for *BRCA1/2*-mutated ovarian cancer, and Rucaparib, approved for ovarian and prostate cancers, are also under evaluation for the treatment of PDAC in patients with known DNA repair mutations [136,137,138]. Veliparib is currently under Phase II investigation in the treatment of PDAC, both as monotherapy and in combination with chemotherapy, having achieved orphan drug status for advanced squamous cell non-small cell lung cancer [139,140].

While PARPis have shown clinical benefit in certain PDAC patients, resistance—both intrinsic and acquired—remains a significant limitation. As a complementary approach, targeting poly (ADP-ribose) glycohydrolase (PARG), the enzyme responsible for degrading poly (ADP-ribose) (PAR) chains, may offer a strategy to overcome PARPi resistance. We have previously published that PARG is an exploitable therapeutic target for PDAC with HR repair gene mutations [141]. IDE161, a first-in-class PARG inhibitor, is currently being evaluated in a Phase I trial in HR-deficient tumors, including advanced and metastatic solid tumors with BARD1 mutations [142]. PARG hydrolyzes PAR chains, which are synthesized by PARPs and serve as recruitment scaffolds for DNA repair proteins. In the absence of a functional PARG, PAR chains accumulate, sequestering DNA repair factors on chromatin and impairing efficient DNA damage resolution. Preclinical studies suggest that this mechanism sensitizes tumor cells to chemotherapy and ionizing radiation, including in settings where PARPis may be ineffective [143].

Other investigational approaches target key DNA damage kinases, including ataxia telangiectasia mutated and Rad3-related kinase (ATR) and its downstream effector checkpoint kinase 1, well-characterized regulators of replication stress responses and cell cycle checkpoints. Several clinical trials assessing ATR inhibitors in PDAC, regardless of DDR mutation status, are currently active [130,144,145]. A related strategy involves inhibition of the tyrosine kinase Wee1, which modulates G2/M transition via phosphorylation of CDK1. Azenosertib (ZN-c3), a Wee1 inhibitor, is currently in Phase I and Phase II trials for PDAC, including a combination trial with Gemcitabine [133,146].

LP-184, a DNA-alkylating prodrug activated by prostaglandin reductase 1, is also under investigation for solid tumors harboring DDR gene mutations, including PDAC [147]. A separate Phase I trial is evaluating MOMA-313, a DNA polymerase θ inhibitor, alone or in combination with Olaparib in patients with HR-deficient tumors, including pancreatic cancer [148]. Notably, *BARD1* mutations are listed as inclusion criteria in several of these trials.

Preclinical evidence also supports a role for direct inhibition of HR in PDAC via interference with the BRCA1-BARD1 complex. BBIT20, a first-in-class disruptor of BRCA1-BARD1 dimerization, was recently shown in vitro to reduce PDAC cell proliferation regardless of *BRCA* mutation status. In Gemcitabine-resistant PDAC cells, BBIT20 reversed resistance by inhibiting the drug efflux pump P-glycoprotein, upregulating the Gemcitabine transporter ENT1 and downregulating Gemcitabine resistance-related miR-20a and Gemcitabine metabolism enzymes RRM1/2. The drug was also shown to inhibit patient-derived PDAC organoid growth and enhance the efficacy of Olaparib and Gemcitabine, supporting its use in combination therapy [149]. Collectively, these investigational strategies underscore the evolving landscape of DDR-targeted therapies in PDAC, with particular promise in overcoming chemoresistance and expanding treatment options beyond BRCA-mutated tumors.

## 9. Conclusions and Perspectives on Research Strategies

BARD1 plays a complex role in cancer biology, functioning as both a tumor suppressor and an oncogene, depending on the expression of isoforms, structural integrity, and regulatory environment. As a binding partner of BRCA1, FL-BARD1 supports homologous recombination repair and genomic stability, while also contributing to apoptotic signaling through both BRCA1-dependent and -independent mechanisms. Disruption of this function, whether through germline or somatic mutations, enhanced degradation, promoter methylation, or loss of key structural domains, has been implicated in tumorigenesis and therapeutic resistance across multiple cancer types, including PDAC. Conversely, numerous alternatively spliced BARD1 isoforms, notably BARD1β and BARD1δ, exhibit oncogenic potential through diverse mechanisms, including disruption of BRCA1 nuclear localization. The expression of these isoforms appears to be regulated by both genetic and epigenetic factors, although these regulatory layers remain incompletely understood in many tumor contexts.

In PDAC, the clinical relevance of both FL-BARD1 and BARD1 isoforms remains insufficiently characterized. Emerging evidence suggests that FL-BARD1 participates in PAR binding and PARP-mediated DNA damage repair [150]; however, further studies will be needed to both support BARD1 modulation as a potential DDR-targeted therapy and clarify how isoform-specific expression patterns may influence the efficacy of synthetic lethality-based approaches. Further, given the divergent roles of BARD1 isoforms in cancer pathogenesis, continued efforts to profile isoform-specific expression and function in PDAC are urgently needed. Genomic profiling of tumor tissue and patient blood could shed light on the diagnostic and prognostic importance of BARD1 in PDAC. Broader implementation of targeted RNA panels that include BARD1 in clinical oncology, combined with long-read sequencing, will enable high-throughput screening of BARD1 expression, including novel isoforms, across patient samples. To further clarify the cellular roles of BARD1 isoforms and interactions with BRCA1, p53, and other regulatory factors, and even their influence on DNA repair inhibitors, isoform-specific overexpression and knockdown, for example, with siRNAs or splice-switching oligonucleotides models, may be used to guide the development of anti-BARD1 combination therapies, not only in PDAC but also other tumor types where BARD1 plays an important role. Additionally, the detection of auto-antibodies against BARD1 epitopes in plasma samples has been successfully demonstrated in cancer patients and may serve as a complementary approach to established biomarkers [151,152].

Future work should also investigate how BARD1 interacts with the tumor immune microenvironment, influences response to targeted immunotherapies, and evolves during tumor progression, including post-translational or post-transcriptional modifications that alter FL- and isoform expression, or interactions with other DNA damage response proteins that may impact tumor behavior and treatment resistance. In conclusion, elucidating the full spectrum of BARD1’s functions, as a tumor suppressor (“friend”) and a pro-cancer agent (“foe”), will be essential for understanding its dualistic role in cancer and harnessing its biology for therapeutic gain.

## Figures and Tables

**Table 1:** Known BARD1 mutations in pancreatic cancer. dnSNP: Single Nucleotide Polymorphism Database; ANK: ankyrin repeat; BRCT: BRCA1 C-terminal.

Variant Type	dbSNP ID	Coding Change	Amino Acid Change	Exon	Protein Domain	ClinVar Classification	Reference
Nonsense	rs762171436	c.632T > A	p.Leu211Ter	4	None	Pathogenic/Likely Pathogenic	Chaffee et al., 2018 [47]
Frameshift	rs28997575	c.1075_1095del	p.Leu359_Pro365del	4	None	Benign/Likely Benign	Amemiya et al., 2015 [65]; Hu et al., 2016 [46]
Missense	rs2229571	c.1134G > C	p.Arg378Ser	4	None	Benign	Hu et al., 2016 [46]; Zhou et al., 2009 [66]
Missense	rs2070094	c.1519G > A	p.Val507Met	6	ANK	Benign/Likely Benign	Sauer et al., 2005 [67]; Hu et al., 2016 [46]
Missense	rs28997576	c.1670G > C	p.Cys557Ser	7	None	Benign	Stacey et al., 2006 [68]; Hu et al., 2016 [46]
Missense	rs730881420	c.1685C > T	p.Thr562Ile	8	None	Uncertain Significance	Hu et al., 2016 [46]
Missense	rs587782279	c.1693C > T	p.Arg565Cys	7	None	Uncertain Significance	Chaffee et al., 2018 [47]
Missense	rs35306212	c.1738G > A	p.Glu580Lys	8	BRCT	Benign/Likely Benign	Hu et al., 2016 [46]
Nonsense	rs587781948	c.1921C > T	p.Arg641Ter	10	BRCT	Pathogenic	Ramus et al., 2015 [69]; De Brakeleer et al., 2016 [70], Hu et al., 2016 [46]
Missense	rs3738888	c.1972C > T	p.Arg658Cys	10	None	Benign/Likely Benign	De Brakeleer et al., 2010 [71]; Klonowska et al., 2015 [72]; Hu et al., 2016 [46]
Missense	rs61754118	c.2212A > G	p.Ile738Val	11	BRCT	Benign/Likely Benign	Sauer et al., 2005 [67]; Gorringe et al., 2008 [73]; Hu et al., 2016 [46]

**Table 2:** A list of clinical trials evaluating DNA damage response agents in the treatment of cancer. PFS: progression-free survival; ORR: objective response rate; OD: optimal dose; RR: response rate; AE: adverse event; DLT: dose-limiting toxicity; SAE: serious adverse event; MTD: maximum tolerated dose; DoR: duration of response; OS: overall survival; TEAE: treatment-emergent adverse event; PDAC: pancreatic ductal adenocarcinoma; NSCLC: non-small cell lung cancer; TNBC: triple-negative breast cancer; SCLC: small cell lung cancer; DDR: DNA damage response; HRD: homologous recombination deficiency; PARG: poly(ADP-ribose) glycohydrolase.

NCT Number	Phase	Interventions (Mechanism of Action)	Patient Population	Primary Endpoint
NCT03601923	II	Niraparib (PARP inhibitor)	Patients with advanced pancreatic adenocarcinoma with mutations in BRCA1, BRCA2, PALB2, CHEK2, or ATM	PFS at 6 months
NCT03553004	II	Niraparib (PARP inhibitor)	Patients with metastatic PDAC exposed to prior chemotherapy with genes involved in DNA repair	ORR at 8 weeks
NCT04550494	II	Talazoparib (PARP inhibitor)	Patients with solid tumors and documented aberrations in DDR-related genes, including BRCA1/2 and BARD1	Percent of patients who demonstrate simultaneous RAD51 activation and lack of γ-H2AX activation, defined as ≥5% of cells with ≥5 RAD51 foci and <4% nuclear area positive for γ-H2AX at the cycle two, day one biopsy
NCT03140670	II	Rucaparib (PARP inhibitor)	Patients with pancreatic adenocarcinoma on platinum-based treatment and documented BRCA1/2 or PALB2 mutation	PFS at 6 months
NCT02677038	II	Olaparib (PARP inhibitor)	Patients with stage IV PDAC with any genetic alterations conferring HRD, with the exception of germline BRCA1/2	ORR at 5 years and 8 months
NCT04666740	II	Pembrolizumab (PD-L1 inhibitor) + Olaparib (PARP inhibitor) in maintenance setting	Patients with metastatic pancreatic adenocarcinoma or acinar cell carcinoma with genetic alterations conferring HRD with stable disease on platinum treatment Note: Patients are stratified into core HR genes (BRCA1/2, PALB2) and non-core HR genes (including BARD1)	PFS at 6 months
NCT02498613	II	Cediranib (VEGF inhibitor) + Olaparib (PARP inhibitor)	Metastatic or unresectable NSCLC, TNBC, PDAC, or SCLC with at least 1 prior line of systemic treatment	ORR at 43 months
NCT01585805	II	Veliparib (PARP inhibitor) vs. Veliparib + Gemcitabine hydrochloride (nucleoside analog) + cisplatin (alkylating agent) vs. Gemcitabine + cisplatin	Metastatic pancreatic adenocarcinoma with BRCA1/2 or PALB2 mutation	OD at 21 days and RR at 5 years
NCT05933265	I/II	LP-184 (alkylating prodrug) vs. LP-184 + spironolactone (mineralocorticoid receptor antagonist) vs. LP-184 + Olaparib (PARP inhibitor), in TNBC subset only	Patients with advanced solid tumors, with a preference for tumor types with high prevalence of DDR gene mutations (TNBC, lung, prostate, ovarian, pancreatic, bladder, and glioblastoma)	Incidence and severity of all AEs, MTD, and recommended Phase II dose at 12 months
NCT06545942	I	MOMA-313 (DNA polymerase θ helicase inhibitor) vs. MOMA-313 + Olaparib (PARP inhibitor)	Patients with advanced or metastatic solid tumors that are not eligible for curative therapy, with any genetic alterations conferring HRD	Number of participants with AEs, DLTs, SAEs, and/or AEs leading to discontinuation
NCT03682289	II	Ceralasertib (ATR kinase inhibitor) vs. ceralasertib + Olaparib (PARP inhibitor) vs. ceralasertib + durvalumab (immunotherapy)	Patients with a solid tumor malignancy with progression on at least one prior systemic therapy, including all pancreatic cancers	ORR at 3 years
NCT06015659	II	ZN-c3 (Wee1 inhibitor) + gemcitabine (nucleoside analog)	Advanced pancreatic adenocarcinoma in the second line	PFS at 6 months
NCT04616534	I	Elimusertib (ATR kinase inhibitor) + gemcitabine (nucleoside analog)	Patients with advanced pancreatic adenocarcinoma or ovarian cancer	MTD, incidence of AEs, ORR, DoR, PFS, and OS at 1 year
NCT02595931	I	Berzosertib/M6620 (ATR kinase inhibitor) + irinotecan (topoisomerase I inhibitor)	Patients with solid tumors that are metastatic or unresectable, with known deficiencies in DDR genes	MTD
NCT05787587	I	IDE-161 (PARG inhibitor) vs. IDE-161 + pembrolizumab (immunotherapy)	Patients with metastatic solid tumors with genetic alterations conferring HRD, including BRCA1/2 and BARD1	Incidence of DLTs, AEs, TEAEs
